# Multiple Meningioma Resection by Bilateral Extended Rostrotentorial Craniotomy with a 3D-Print Guide in a Cat

**DOI:** 10.3390/vetsci9100512

**Published:** 2022-09-20

**Authors:** Kyohyuk Song, Haebeom Lee, Jaemin Jeong, Yoonho Roh

**Affiliations:** 1 Department of Veterinary Surgery, College of Veterinary Medicine, Chungnam National University, Daejeon 34134, Korea; 2 Division of Small Animal Surgery, Department of Clinical Veterinary Medicine, Vetsuisse-Faculty University of Bern, 63012 Bern, Switzerland

**Keywords:** meningioma, multiple meningiomas, craniotomy, 3D-guide, feline

## Abstract

**Simple Summary:**

Meningioma is the most common intracranial neoplasia in cats. Treatments for meningiomas—including complete surgical resection, debulking, irradiation, or palliative therapy—have been reported in veterinary medicine. However, multiple meningiomas (two or more meningiomas in the same patient, separated by anatomical location) have been reported to affect the complication rate and prognosis. Moreover, the characteristics of neurosurgery—such as accurate localization and awareness of the anatomical structures of the lesions—make the surgery especially difficult for inexperienced surgeons. Surgical navigation systems have been developed, but recently, patient-specific three-dimensional(3D)-printed models and guides have also been used in orthopedics and neurosurgeries for treating many disorders with good results. A 13-year-old castrated male domestic shorthair cat was referred with multiple meningiomas located within the right frontal and occipital lobes. The cat suffered from generalized tonic–clonic seizures and mild proprioceptive ataxia. After removing both of the tumors, the cat showed a favorable clinical outcome and no neurological abnormalities throughout long-term follow-up. With a patient-specific 3D guide technology, a craniotomy for multiple meningiomas can be performed safely and accurately.

**Abstract:**

A 13-year-old castrated male domestic shorthair cat was referred for the surgical removal of multiple meningiomas. The cat experienced generalized tonic–clonic seizures, altered mentation, mild proprioceptive ataxia, and circling. Magnetic resonance imaging (MRI) revealed two round, solitary, well-delineated, space-occupying lesions suggestive of multiple meningiomas in the right frontal and occipital lobes. Before surgery, patient-specific three-dimensional (3D) printed models and guides were produced using a 3D program based on MRI and computed tomography (CT), and a rehearsal surgery was performed. With a 3D guide to find the location of the craniotomy lines, bilateral extended rostrotentorial craniotomy allowed en bloc resection of multiple meningiomas. The bone fragment was replaced and secured to the skull with a craniofacial plate and screws with an artificial dura. All of the surgical steps were performed without complications. The preoperative presenting signs were resolved by the time of follow-up examinations 2 weeks after surgery. Twelve months after the removal of the multiple meningiomas, the cat survived without further neurological progression. For the resection of multiple meningiomas, surgery can result in large bone defects and risk of massive hemorrhage. For this challenging surgery, patient-specific 3D models and guides can be effective for accurate and safe craniotomies.

## 1. Introduction

The most common intracranial neoplasia in cats is meningioma [1]. In a recent retrospective review of intracranial neoplasia in cats, meningioma was reported in approximately 58.1% of cases of feline intracranial neoplasia [1,2]. In addition, multiple meningiomas occupy a large place among meningioma cases, with an occurrence rate of approximately 17% [1,2,3].

Most intracranial meningiomas are extra-axial intracranial tumors that originate from arachnoid cap cells, which are cells of the arachnoid villi in contact with the venous endothelium of the venous sinus of the dura [2,4,5]. These tumors frequently develop in the third ventricle and supratentorial regions, such as the parietal, temporal, and occipital lobe [1]. Multiple meningiomas represent two or more meningiomas in the same patient, separated by anatomical location [6]. Three theories have been proposed to account for the etiology and pathogenesis of multiple meningiomas: multicentric dural foci; metastasis by blood-borne spread; and metastasis by the cerebrospinal fluid. Due to the tumor being histologically benign and the report showing histological variants in the same patient, the first theory is more supportive than the other two [3,7].

Treatment options for meningiomas include complete surgical resection, debulking, irradiation, or palliative therapy. In previous reports, each treatment option showed significant differences in the median survival time. The median survival time for cats following various treatments were as follows: surgical resection, 26 months; irradiation therapy, 14 months; and medical management, 18 days [1,8,9]. Unlike canine meningiomas, feline intracranial meningiomas are usually benign, fibrotic, and well encapsulated, and surgical excision is the standard treatment [1,5]. In the case of multiple meningiomas, which have characteristics similar to solitary meningiomas, the number of meningiomas does not have a significant effect on the survival and outcome of surgery if they have been assessed correctly and surgical resection has been performed thoroughly [2,6]. However, surgical resection of multiple meningiomas is associated with complications; complicated surgical plan, prolonged surgical time, intraoperative hemorrhage, and limited surgical space leading to brain parenchymal traction injury [8,10]. To prevent these complications and decrease mortality and morbidity in neurosurgery, accurate localization, proper size and shape of craniotomy, and awareness of the anatomical structures adjacent to the lesions are essential [10,11,12]. However, especially for inexperienced surgeons, it is difficult to determine the correlation between the intraoperative three-dimensional (3D) space and surgical plan from two-dimensional (2D) orthogonal images [12].

Various techniques have been developed, including surgical navigation systems (SNSs) and 3D printing models and guides. In human medicine, SNSs have been proven beneficial in intracranial surgery [13], and they can help surgeons optimize access to the lesion by providing information on anatomo-pathological relationships [13]. However, there are some difficulties in using SNSs in clinical veterinary medicine, such as anatomical variability between species, high expense, and prolonged operative time [11,14].

Recently, the use of 3D-printed models and guides has also increased in human and veterinary medicine to overcome these limitations. The use of 3D-printed surgery models help surgical teams carry out rehearsal surgery, patient-specific surgical planning, and identification of the exact intraoperative location of the lesion [12,15]. It also has the benefits of time saving, better surgical outcomes, and low cost [15,16]. However, to the best of our knowledge, there are few reports on the use of patient-specific 3D-guides and models for multiple meningioma resections in veterinary medicine. We describe a case of craniotomy for the removal of multiple meningiomas in an aged cat using a patient-specific 3D-guide and rehearsal surgery for successful outcome and discuss the specific techniques to perform multiple meningioma surgeries and prevent complications.

## 2. Case Presentation

### 2.1. Case

A 13-year-old castrated male domestic shorthair cat was referred for surgical removal of presumed multiple meningiomas within the right frontal and occipital lobe detected during magnetic resonance imaging (MRI) at the referring veterinary clinic. Reported clinical signs included generalized tonic–clonic seizures, altered mentation, and circling. In addition, the patient was not able to jump or climb to higher places. The cat was diagnosed with multiple meningiomas 2 months before referral and was initially treated with prednisolone (0.5 mg/kg PO q12h) and hydroxyurea (25 mg/kg SID) for two months.

**Figure 1:**
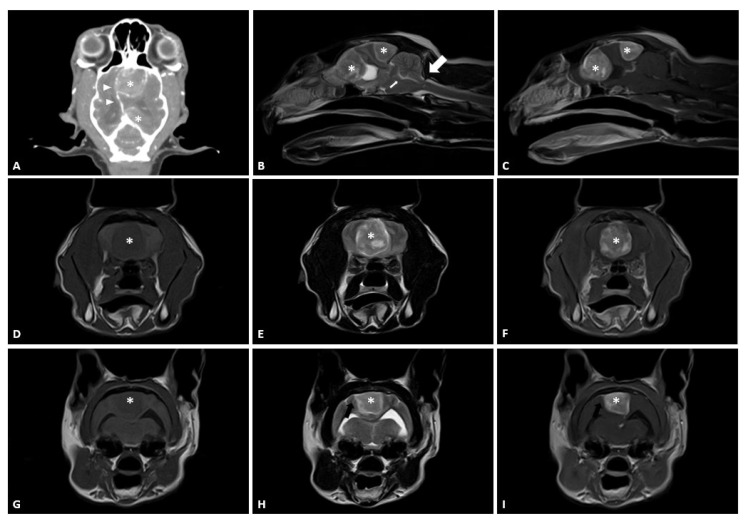
Preoperative post-contrast computed tomography (CT) and magnetic resonance images (MRI) of the head and cervical area. (**A**) Dorsal post-contrast CT images. Contrast-enhanced space-occupying lesions (star) are observed. A brain midline shift (arrowhead) was identified. (**B**) Midline sagittal T2-weighted (T2W) images. Hyperintense space-occupying lesion (star) creates transtentorial herniation (small arrow) and cerebellar herniation (large arrow). (**C**) Midline sagittal T1-weighted (T1W) post-contrast images. Strongly contrast-enhanced heterogenous space-occupying lesions (star) are observed. (**D**,**G**) Transverse T1W images at the frontal and occipital lobe. Hypointense space-occupying lesions (star) are observed. (**E**,**H**) Transverse T2W images at the frontal and occipital lobe. Hyperintense space-occupying lesions (star) are observed. (**F**,**I**) Transverse T1W post-contrast images at frontal and occipital lobe. Strongly contrast-enhanced heterogenous space-occupying lesions (star) are observed. The dural tail sign (black arrow) is identified adjacent to the mass at the occipital lobe.

At presentation, the patient exhibited circling and ataxia. MRI showed a round, solitary, well-delineated, space-occupying lesion with marked hypointense to isointense T1-weighted (T1W) images, hyperintense T2-weighted (T2W) images, and strong contrast-enhanced T1W post-contrast images in the right frontal and occipital lobe (Figure 1). It also showed concurrent brain edema and mass effect, midline shift of brain, transtentorial herniation, and cerebellar herniation. These transtentorial and cerebellar herniations may represent signs of increased intracranial pressure [17]. The dural tail sign was also evident on MRI images with a thickening and enhancement of the dura adjacent to the mass, which is characteristic of an extra-axial mass. This strongly suggested that the masses were meningiomas. The masses had the same characteristics. Thoracic radiography and abdominal ultrasonography did not reveal any abnormalities and evidence of metastasis. Hematology and blood chemistry results were within normal limits.

### 2.2. Preoperative Planning and Rehearsal Surgery

**Figure 2:**
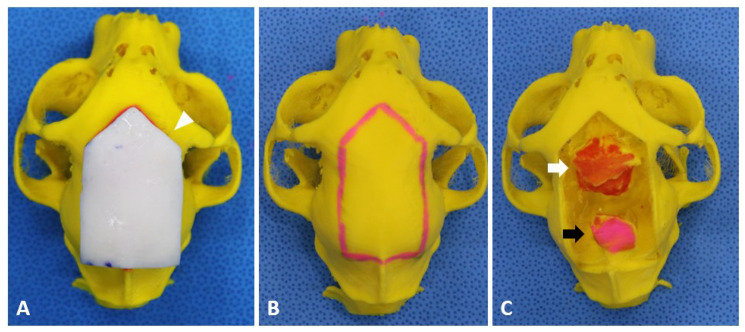
Preoperatively designed three-dimensional (3D) guide and models for rehearsal surgery and intraoperative craniotomy. (**A**) 3D-printed guide (arrowhead) is produced to determine the extended rostrotentorial craniotomy line by fitting on to the skull. (**B**) The 3D model represents the craniotomy line (red line). (**C**) 3D model represents the location of meningiomas in the frontal (white arrow) and occipital lobes (black arrows).

Surgical planning involving confirmation of the location, size, and shape of the craniotomy line and identifying the exact location of the tumor was performed under computer-aided 3D design software (Mimics, Materialise NV, Leuven, Belgium) based on MRI and CT images. In addition, 3D models were produced and obtained according to the program and a previously described protocol [16]. A 3D craniotomy guide was designed to provide an exact craniotomy line during surgery by press-fit to the patient’s cranium (Figure 2A) [16,18]. It was decided that the craniotomy line was to be larger than traditional craniotomy methods because of the tumor size. The craniotomy line was determined by planning a bilateral extended rostrotentorial craniotomy. A bilateral extended rostrotentorial craniotomy indicates a bilateral and rostrotentorial craniotomy comprised of two bilateral craniotomies over the sagittal sinus. A bilateral extended rostrotentorial craniotomy contains craniotomies to the frontal, parietal, and occipital bone, bilaterally over the sagittal crest. At the cranial border, the craniotomy was planned trans-frontally without opening the frontal sinus. For the caudal part of the craniotomy, the craniotomy was determined trans-occipitally not to cross the border of the transverse sinus without exposing the sinus. For the lateral aspects, the line determined for the craniotomy was 1.2–3 times wider than the tumor to remove the tumor without excessive traction of the brain parenchyma. After confirming the location of the tumor and the craniotomy line, a patient-specific 3D guide was designed to fit onto the skull to find the craniotomy line. For the guide to fit perfectly onto the skull, its contact surface was designed to express the inverted contact area of the skull [18]. The bone models and patient-specific 3D guide were produced using a fused deposition modeling 3D printer (Cubicon style NEO-A22C (Cubicon Inc., Gyeonggi-do, Korea)) and a resin-based 3D printer (Photon mono X 6 K (Anycubic, Shenzhen, China); Figure 2).

After producing the patient-specific 3D models and guides, the rehearsal surgery was performed to familiarize the surgeon with the surgical procedure and patient-specific anatomo-pathological features. In addition, the ideal position of the patient for the procedure—i.e., sternal recumbency with the head elevated to approximately 30° without lateral tilting to promote brain “dropping” with the aid of gravity—was determined. After the rehearsal surgery, the 3D models and guide were sterilized with plasma sterilizer (RENO-S30 low temperature plasma H_2_O_2_ gas sterilizer (RENOSEM, Gyeonggi-do, Korea)).

### 2.3. Surgical Techniques

For anesthetic premedication and induction, Midazolam (0.2 mg/kg, IV (Midazolam^®^, Bukwang pharmaceutical, Seoul, Korea)), Medetomidine (1 µg/kg IV (Tomidine; Provet, Istanbul, Turkey)), Dexamethasone (0.2 mg/kg, IV; (Dexamethasone; Jeil Pharm. co., Ltd., Daegu, Korea)), and Alfaxalone (3 mg/kg, IV; (Alfaxan^®^ multidose, Jurox, West Sussex, UK)) were administered [8]. Antibiotic prophylaxis was initiated with administration of Cefazolin (22 mg/kg, IV (Cefazolin, Jong Geun Dang, Seoul, Korea)) [8]. Remifentanil (5–10 µg/kg, Continuous rate infusion (CRI); (Remiva^®^, Hana Pharm, Seoul, Korea)) was used for analgesia. Anesthesia was maintained with isoflurane (0.7–0.8% (Ifran^®^, Hana Pharm, Seoul, Korea)). Plasmalyte (7.5 mL/kg/h CRI (Plasma Solution-A^®^, HK inno.N, Seoul, Korea)) was administered throughout the procedure. Mannitol (0.5 g/kg slow IV (D-Mannitol inj. K.P., DaiHan Pharm Co., Ltd., Seoul, Korea)) was also administered to relieve increased intracranial pressure (ICP) and to induce brain relaxation [19,20].

The surgical site was clipped from the lateral canthus of the eyes to the occipital condyle, and laterally to the zygomatic arches. The skin was prepared aseptically, and the cat was placed in sternal recumbency with the head elevated by approximately 30° without tilting. The incision line was determined based on the patient-specific 3D-printed models and a guide. A linear incision was made on the skin at the midline, extending from the caudal end of the nasal bone to the external occipital protuberance. The subcutis, fascia, and periosteum were bluntly retracted using a periosteal elevator.

**Figure 3:**
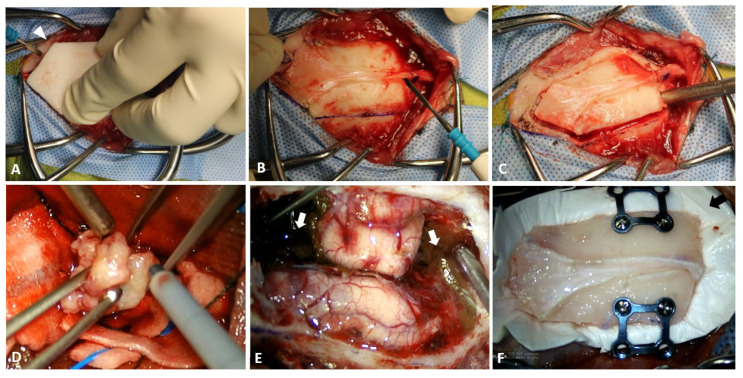
Application of three-dimensional (3D)-print guide (arrowhead) and surgical procedure. (**A**) Application of 3D-print guide. After removing soft tissues attached to the frontal, parietal, and occipital bone, the 3D-print guide is placed on the calvaria. (**B**) The osteotomy line is marked with electrocautery following the 3D-print guide. (**C**) The bilateral extended rostrotentorial craniotomy is performed with a 2.0 mm burr and fine Kerrison rongeur. (**D**) Removal of meningioma is performed by ultrasonic surgical aspirator for en bloc resection with open-window technique of the meningioma. (**E**) After removing the meningiomas, to control hemorrhage, hemostatic gelatin sponge and hemostatic matrix (white arrow) are used. (**F**) Closure of the cranium. To prevent cerebrospinal fluid leakage, artificial dura (black arrow) is used. The bone fragment is placed and secured to the skull with craniofacial plate and screws.

Craniotomy was planned using the specific 3D-printed guide to conform to the craniotomy line (Figure 3A). The craniotomy line was drawn using a surgical pen and electrocautery (Figure 3B). The osteotomy was completed using a 2.0 mm bone burr (Stryker Corp., Kalamazoo, MI, USA) and fine Kerrison rongeur (Figure 3C). The cut created a shield-shaped bone fragment that was removed using a periosteal elevator. Despite careful dissection of the dura and periosteum, the dura mater tore while the bone fragment was being removed. The sagittal sinus was intact. The sagittal sinus deviated ventro-laterally secondary to the large volume of the meningioma. The inner surface of the bone fragment was debrided with a 2 mm bone burr and rongeur to remove the attached tissues. The bone fragment was stored in wet gauze with saline for replacement at the end of the procedure.

After the craniotomy, the masses were gently mobilized by grabbing their surface with atraumatic forceps and packing lint-free gauze between the tumor and brain tissues to divide the tumor from the brain parenchyma. En bloc resection was performed with the open-window technique by enucleating the tumor with tumor forceps or surgical aspirator (Sono Cure (Anchor Medical Products LLC, Coatesville, PA, USA); Figure 3D). After enucleating the tumor, the tumor capsule was carefully dissected from the brain parenchyma [21]. The masses were submitted for histological examination. During the en bloc resection of the meningiomas, hemorrhage from the excisional area was controlled using bipolar cauterization, lint-free gauze, hemostatic gelatin sponge (Spongostan (Ethicon, Raritan, NJ, USA)), and hemostatic matrix (Floseal^®^ (Baxter Healthcare Corporation, Fremout, CA, USA); Figure 3E).

After the en bloc resection of the meningiomas, an oozing hemorrhage was observed. The hemostatic matrix was applied twice for each surgical site to control oozing hemorrhage from the surgical site. The application time for each process was 2 min, following which all matrixes were flushed out with physiological saline, and the hemorrhage was evaluated again [22]. When hemostasis was achieved at all surgical sites, the site was flushed with physiological saline. To prevent cerebrospinal fluid (CSF) leakage, the dural defect was covered by an oversized artificial dura (Redura (Medprin Biotech GmbH, Germany)) and the bone fragment was fixed with a 0.6 mm craniofacial plate and screws (Jeil Medical Co., Seoul, Korea). The craniofacial plate was pre-contoured using the patient-specific 3D model. The fascia and subcutis were sutured separately and continuously with polydioxanone (PDS) 4-0 and Monocryl 4-0, monofilament absorbable suture. The skin was closed using a skin stapler.

### 2.4. Postoperative Care, Outcomes, and Pathological Examination

Postoperatively, the cat was sedated for the first 24 h with medetomidine (1 ug/kg/h CRI (Tomidine; Provet, Turkey) [23]. The cat received prophylactic antibiotics for 10 days with cefotaxime (20 mg/kg IV BID (Cefotaxim, Hankook Korus, Kang-won do, Korea)) and clindamycin (11 mg/kg IV BID (Fullgram^®^; Samjin Pharm. Co., Ltd., Seoul, Korea)). The cat also received dexamethasone (0.1 mg/kg IV SID, (Dexamethasone; Jeil Pharm. Co., Ltd., Seoul, Korea)) for 3 days to prevent surgical site swelling after surgery. Phenobarbital (1.5 mg/kg IV BID, (Phenobarbital inj., Jeil Pharm. Co., Ltd., Seoul, Korea)), and Levetiracetam (20 mg/kg IV q8h, (Keppra; UCB Pharma S.A., Brussels, Belgium)) were also administered after surgery.

On the day after surgery, the cat showed no abnormalities on physical examination. Neurological examination revealed mild proprioceptive ataxia with mildly dragging hind limbs. Menace response and pupillary light reflex were normalized just a day after surgery. The preoperative presenting signs were resolved by the time of the 2-week follow-up examination. The cat was discharged from the hospital with a normal gait and without any neurological abnormalities. Telephonic follow-ups were conducted to obtain information regarding patient status and gait evaluation. Clinical follow-up could not be performed because of the difficulties faced by the owner in visiting the hospital with the cat.

At 370 days after the operation, the cat showed alert mentation, without further complications such as neurological progression or ataxia. In addition, the cat could jump and climb higher than it could before surgery. Further postoperative MRI and CT were denied by the owner due to the risk of repeated anesthesia for the patient and costs.

Postoperative histological diagnosis was meningioma for both the masses. The two types of samples exhibited the following similar characteristics: moderate to dense cellularity and spindle cells arranged in bundles and a few whorls. A small amount of collagen was observed. The cells had a moderate amount of cytoplasm and elongated nuclei. The mitotic count was 1 mitoses/10 HPF. The tumors were classified as grade 1 (benign meningioma) fibroblastic subtype based on the human World Health Organization schemes, which is one of the most common subtypes of feline meningiomas [24].

## 3. Discussion

To the best of our knowledge, this is the first report to describe the resection of multiple meningiomas by bilateral extended rostrotentorial craniotomy using a patient-specific 3D guide. The 3D-printed guide perfectly fit the skull as designed, and the craniotomy was completed as planned. The accurate surgical approach makes the surgical procedure safer. The patient had excellent outcome for a long-term follow-up of 370 days without complications. The cat showed improvement in ataxia and reaction to external stimuli without seizure or ataxia after complete en bloc resection of meningiomas using the open-window technique.

In human medicine, if the total resection of meningioma presents risks of further complications because of the location or adjacent anatomic structures of meningioma, subtotal resection or debulking is inevitably performed rather than complete resection [3,4]. However, the limited extent of resection of the tumor and surrounding tissues related to anatomical features regularly leads to early tumor recurrence. In previous reports in human medicine, the recurrence rate after complete resection of the affected dura and bone was 5%, and the recurrence-free rate 5 years after complete resection was 93%. However, the recurrence rate after subtotal resection was 35%, and the progression-free rate 5 years after surgery was only 63% [25,26]. In previous reports in feline meningiomas, the recurrence rate after resection of meningiomas were approximately 20% [1,2,5]. However, to the best of our knowledge, there are no studies comparing the recurrence rate according to the surgical margins in feline meningioma. However, complete resection is the theoretical goal of meningioma surgery to prevent recurrence. Feline meningiomas are also expected to have a good prognosis with en bloc resection rather than debulking or subtotal resection. In addition, feline intracranial meningiomas are usually benign, fibrotic, and well-encapsulated [1,2,5]. For these reasons, en bloc resection could be the first choice for surgical decisions in meningioma cases. In this case, en bloc resection by adequate craniotomy using a 3D-printed guide was performed, and the outcome of the surgery was good with no further seizures or need for administration of anticonvulsant drugs.

Various surgical approaches and craniotomy sites are chosen based on the anatomical location of the meningioma and patient-specific considerations [2,4,21]. Furthermore, it is crucial to ensure an excellent approach to the lesion because it facilitates good exposure of the lesion to decrease the risk of iatrogenic injury of the brain parenchyma from unnecessary traction and manipulation [27,28]. Extended bilateral craniotomy was planned in this case because the meningiomas had a large volume for total removal without excessive traction and manipulation of the brain parenchyma in unilateral craniotomy. In addition to the large meningioma in the frontal lobe, there was an additional meningioma in the occipital lobe. Although additional craniotomies have been used according to some reports, these additional craniotomies present a risk of prolonged operative time, sinus injury, and retraction injuries to the brain [29,30]. Hence, the bilateral extended rostrotentorial craniotomy—a wide bilateral single craniotomy from frontal bone to occipital bone—was planned for en bloc resection of the multiple meningiomas in this case.

In addition to an excellent craniotomy plan that determines the craniotomy localization, size, and shape, accurate craniotomy and determining anatomo-pathological relationships are crucial in neurosurgical procedures to reduce mortality and morbidity [10,13]. An accurate craniotomy is also necessary to sufficiently expose the surgical sites for tumor resection, without excessive manipulation of the brain parenchyma. However, it is difficult for surgeons—especially novices—to precisely identify the location and shape of the craniotomy site and anatomo-pathological relationships using only bone landmarks based on 2D CT or MRI [10,12,31]. By using 3D-printing methods, patient-specific models enhance the recognition of anatomo-pathological relationships, increase the accuracy of craniotomy localization, and allow pre-surgical rehearsal surgeries [32]. In addition, it is practical and cost-effective [31,33]. In this case, using 3D-printed models and guides, the rehearsal surgery enhanced the recognition of anatomo-pathological relationships and confirmed the surgical procedures. Furthermore, exact craniotomy using a 3D-printed guide helps complete en bloc resection of multiple meningiomas and makes surgical manipulation of meningiomas easier for the surgeon by proper surgical site presentation. In addition, using a 3D-printed guide and rehearsal surgery, the exact craniotomy site was created as planned by the 3D program. It also improves surgical accuracy and safety and reduces surgical time.

Although bilateral extended craniotomy has advantages in reducing excessive manipulation and traction of brain parenchyma, it still carries the risk of damaging the sagittal sinus and collateral and bridging veins. In this case, according to the diagnostic images, the dorsal sagittal sinus was deviated ventro-laterally as a result of the large volume of the meningioma. Furthermore, there was no significant sign of infiltration of meningioma into the dorsal sagittal sinus. These were believed to be positive factors for enhancing the surgical outcome. However, it is still challenging for the surgeon to perform bilateral extended craniotomy while preserving the dorsal sagittal sinus. To reduce the risk of dural tear and iatrogenic injury to the dorsal sagittal sinus and improve surgical outcome, brain relaxation was performed perioperatively [20]. Brain relaxation is a crucial procedure for softening a tight or swollen brain to facilitate surgery while protecting the brain from the traction or compression injuries [20]. There are several management techniques for brain relaxation, such as hyperosmolar therapy, hyperventilation, cerebrospinal fluid drainage, head-up positioning, and steroids [19,20]. In this case, administration of mannitol as a hyperosmolar therapy, hyperventilation before craniotomy, head-up positioning, and administration of dexamethasone as a steroid therapy were used.

In this case, despite the effort to preserve dura mater, a dural tear occurred during the craniotomy. In human medicine, the incidence of dural tears during craniotomy ranges from 20% to 30%, and may include single or both layers of the dura [34,35]. These dural tears frequently occur when the patient has factors that alter dural strength and thickness and increase the adhesion of the dura to the bone, such as meningiomas and hyperostosis frontalis [35]. These defects could cause the patient to undergo an extended period of hospitalization with complications, such as CSF leakage, potential wound infection, cerebral herniation, hypertensive pneumocephalus, and pseudomeningocele [35,36]. To prevent these complications, water-tight closure is a traditional and essential procedure [36]. Traditionally, a simple interrupted suture pattern has been used for primary closure of the dura itself, or other dural substitutes. However, closing the dura primarily by suture alone is difficult because of shrinkage and loss of the native dura mater and it may not be watertight. Furthermore, the dura mater could be torn while implanting synthetic graft materials by suturing [36]. In this case, there was a lack of dura mater for suturing with an artificial dura. To prevent complications from insufficient water-tight closure, an artificial dura, the excised bone fragment, and craniofacial plate and screws were placed similarly to a gasket-seal closure rather than exposing more dura with the burr due to the prolonged surgical time and widening of the craniotomy [37]. No clinical signs of CSF leakage, seroma, or wound infection were observed after surgery.

Traditionally, in human medicine in the Simpson’s grading system of surgery of meningioma resection, the recurrence rate correlates with the extent of adjacent structures removed, including dural attachment, adjacent venous sinus, and nearby hyperostotic bone [38,39,40]. As there are some reports of tumor invasion into the region of hyperostosis, it is also usually a safe and wise decision for surgeons to eliminate all of the affected bony structures to be relieved from the risk of recurrence after resection of meningioma [41,42]. Moreover, in veterinary medicine, removing all of the adjacent and affected hyperostotic bones in meningioma surgery has been considered the golden standard [42]. However, in recent studies, while maximal safe resection is still the main principle of meningioma resection, the validity of Simpson’s grade in relation with recurrence rate has been questioned [43,44]. Furthermore, in a recent study in human medicine, hyperostotic bone that may be affected by infiltration of tumor could be reimplanted with the process of refashioning by drilling off the hyperostotic parts. There were only two cases of recurrence over 5 years secondary to the incomplete tumor resection over the dural sinus wall that were not related to hyperostotic bone fragment [45]. In our case, according to the CT and MRI images, there was no significant sign of hyperostosis and metastasis. For these reasons, reconstruction of the defect was performed with bone fragments with drilling off the attached area and fixing with craniofacial plate and screws.

Our case report has limitations regarding follow-up data because the owner did not want further diagnostic testing. It is essential to perform serial follow-up diagnostic imaging with neurological examinations to accurately assess surgical outcomes and signs of recurrence. If MRI and CT images could have been taken postoperatively, the relationship between the dura mater, plates, and artificial dura could have been assessed. In addition, although this case report showed the effectiveness of a patient-specific 3D-guide for feline meningioma resection, a case series or a prospective study is necessary to confirm the effectiveness and safety. Furthermore, rehearsal surgery with patient-specific 3D models also presents limitations because the models are based on preoperative images. Therefore, they cannot reflect the intraoperative brain distortion or surface bulging after dural opening.

## 4. Conclusions

In this case, patient-specific 3D-printing technology contributed to favorable surgical outcomes in en bloc resection of multiple meningioma. Rehearsal surgery with patient-specific 3D models makes surgeons more familiar with anatomo-pathological relationships and accurate craniotomy localization. With a patient-specific 3D guide, a craniotomy can be performed safely and more accurately. Clear and adequate exposure of the lesion prevents excessive manipulation and traction of the brain parenchyma, resulting in successful en bloc resection of multiple meningiomas using the open-window technique in cats. Patient-specific 3D guides and models may also improve the surgical outcomes of other craniotomies.

## Data Availability

Not applicable.
